# Lymphatics in Eye Fluid Homeostasis: Minor Contributors or Significant Actors?

**DOI:** 10.3390/biology10070582

**Published:** 2021-06-25

**Authors:** Mariela Subileau, Daniel Vittet

**Affiliations:** Biosanté Laboratory UMR 1292, Interdisciplinary Research Institute of Grenoble, University of Grenoble Alpes, Inserm, CEA, 38000 Grenoble, France; mariela.subileau@cea.fr

**Keywords:** lymphatic vessel, glymphatic system, aqueous humor drainage, intraocular pressure, eye disease, glaucoma

## Abstract

**Simple Summary:**

The eye contains fluid compartments whose tight regulation is essential for proper eye functioning. Lymphatic vessels are known to be important in several physiological functions including interstitial fluid homeostasis. Recent works has provided evidence of a potential role of lymphatic vessels in the drainage of ocular fluids. They may in particular contribute to the intraocular pressure regulation, whose increase is a major risk factor for the progression of glaucoma, an eye disease that can lead to blindness. They could also participate in the clearance of toxic waste products responsible for eye retinal neurodegenerative processes. In this review, we performed an update of ocular lymphatic vessel distribution, of their mechanisms of development and of their role in aqueous humor drainage and eye fluid regulation. We discussed these data and made some proposals to elucidate unresolved questions to improve knowledge concerning the lymphatic vessels roles in ocular fluid regulation, which could have repercussions in eye disease therapeutic strategies.

**Abstract:**

Lymphatic vessels exert major effects on the maintenance of interstitial fluid homeostasis, immune cell trafficking, lipid absorption, tumor progression and metastasis. Recently, novel functional roles for the lymphatic vasculature have emerged, which can be associated with pathological situations. Among them, lymphatics have been proposed to participate in eye aqueous humor drainage, with potential consequences on intraocular pressure, a main risk factor for progression of glaucoma disease. In this review, after the description of eye fluid dynamics, we provide an update on the data concerning the distribution of ocular lymphatics. Particular attention is given to the results of investigations allowing the three dimensional visualization of the ocular surface vasculature, and to the molecular mechanisms that have been characterized to regulate ocular lymphatic vessel development. The studies concerning the potential role of lymphatics in aqueous humor outflow are reported and discussed. We also considered the novel studies mentioning the existence of an ocular glymphatic system which may have, in connection with lymphatics, important repercussions in retinal clearance and in diseases affecting the eye posterior segment. Some remaining unsolved questions and new directions to explore are proposed to improve the knowledge about both lymphatic and glymphatic system interactions with eye fluid homeostasis.

## 1. Introduction

The lymphatic vasculature has been recognized to be a key actor in several physiological processes and in many human diseases. Among its functions, the lymphatic vascular system exerts a major role in the regulation of interstitial fluid homeostasis. In addition to the exchanges between plasma and extracellular fluids by the blood vascular system, the lymphatics drain the excess of interstitial fluids that are not returned directly back to the blood capillaries. Extracellular fluids, leukocytes, proteins and metabolites can enter blind-ended permeable lymphatic capillaries to constitute the lymph. Thereafter, by a network of collecting vessels, the lymphatic vasculature transports the lymph through lymph nodes and then back into the venous circulation [1,2]. Beyond interstitial fluid homeostasis, the lymphatic system is essential for immunosurveillance, since it allows immune cell trafficking towards lymph nodes where the immune responses are initiated. Another important role of the lymphatic vessels of the intestinal villi, also called lacteals, concerns lipid absorption [1,2]. Lymphatic vessel involvement in several pathophysiological processes has been shown. Lymphatic vascular deficiency is the cause of both primary and secondary lymphedema, which are characterized by swelling of the extremities due to defective interstitial fluid drainage. Lymphatic vessels also constitute an important facilitator for tumor progression by allowing the dissemination of metastases. More recently, lymphatics have been shown to contribute to organ-specific specialized functions, and to be associated with diseases affecting these organs [1,2].

The eye of mammals holds fluid compartments whose tight regulation is critical to ensure proper eye functions [3]. In addition to the known pathways involved in ocular fluids homeostasis, which will be briefly described in the next chapters, the lymphatic system has been postulated to constitute a novel actor in the drainage of ocular fluids. Several well-documented reviews about research work on eye lymphangiogenesis and describing eye lymphatic vessel distribution have previously been published [4,5,6]. The existence of a rich ocular surface lymphatic vessel network is clearly established. Recently introduced light sheet fluorescence microscopy (LSFM) imaging has allowed its three dimensional visualization in the intact eyeball, providing novel insights of the eye surface lymphatic vessel network distribution. Some works also postulated the existence of some lymphatics in the internal eye structures, although this remains highly debated. Moreover, several experimental results are in favor of a role of these lymphatics in the regulation of eye fluids dynamics, allowing us to consider the lymphatic vasculature as a potential new target for therapeutic strategies in some eye diseases, such as glaucoma. Our goal, in this review, is to provide an up to date analysis of ocular lymphatics and of molecular regulatory pathways identified in their development, and to focus on the recent major advances and discoveries concerning the involvement of lymphatic vessels in the control of eye fluid homeostasis. We will further discuss the reported results and point out unsolved questions that could be of interest in eye diseases linked to aqueous humor drainage, intraocular pressure regulation and/or clearance of toxic waste metabolic products from the eye. The occurrence and the potential interactions with the newly proposed paravascular glymphatic system will also be considered.

## 2. The Fluid Compartments of the Eye and the Ocular Fluids Dynamic

**Figure 1:**
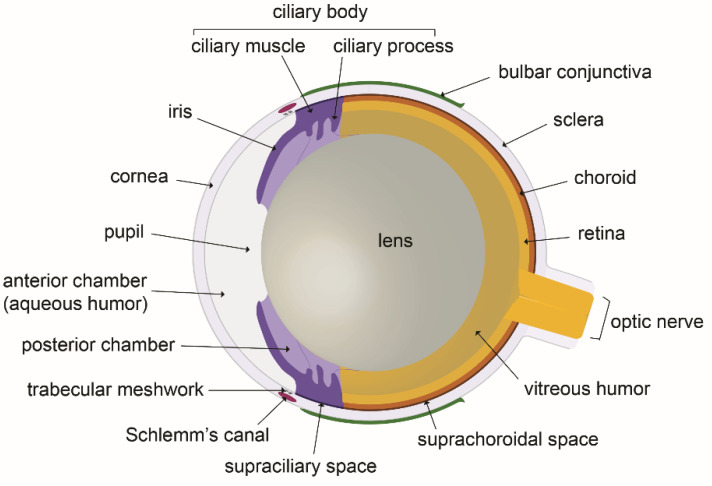
The eye anatomy: schematic illustration of a mouse eye cross section.

As stated above, eye anatomy and proper function needs the maintenance of fluid homeostasis [3]. In the eye of mammals (Figure 1), the spaces between the cornea and the lens in the eye anterior segment are filled by aqueous humor, a transparent biological fluid. Aqueous humor is produced and secreted by the ciliary processes into the posterior chamber from where it moves to the anterior chamber after flowing through the pupil [3,7,8]. Aqueous humor formation results from a complex process of passive and active mechanisms which combine diffusion, filtration and secretion. Plasma diffusion and ultrafiltration through ciliary body fenestrated capillaries, determine the passage into the ciliary stroma of water, large molecules and solutes, depending on ionic concentrations gradients and on both hydrostatic and oncotic pressures. Secretion of aqueous humor into the posterior chamber is further achieved by fluid transfer through the ciliary epithelial cell bilayer. The active ion transport by the outer non-pigmented ciliary epithelial cell layer sets the aqueous humor ionic composition and generates the osmotic pressure gradient which constitutes the driving force at the origin of the secretion process [7,8]. On the other hand, vitreous humor, which constitutes the largest part of the human eye, fills the space between the lens and the retina in the posterior eye segment [9,10]. It displays a viscoelastic gel-like structure due to the presence of various polysaccharides and to a highly hydrated extracellular matrix protein content [9]. Unlike aqueous humor, the vitreous content is not renewed by a permanent outflow. However, some remodeling continuously occurs after birth and during aging, leading to its partial liquefaction [10].

Both aqueous and vitreous humors are involved in proper eye functioning, through the nourishment of the different ocular tissues, the clearance of waste metabolic products, intraocular oxygen concentration regulation and the creation of an internal pressure appropriate for the maintenance of the shape of the eyeball [9,11,12]. Overall, eyeball shape maintenance is essential for the alignment of internal eye components for proper optical transmission and focus of the light signals on the retina. In this context, the control of aqueous humor volume more specifically helps to maintain the convex shape of the cornea [13], whereas the gelatinous consistency of the vitreous humor provides support to the lens and stabilizes the retinal layers [9,12].

The dysfunction of the mechanisms tightly regulating eye fluid movements can be the cause of severe pathologies mostly found in the elderly people. In particular, the aqueous humor fluid dynamics is involved in intraocular pressure (IOP) regulation, whose chronic increase constitutes a major risk factor for the development and the progression of glaucoma [14]. The balance between aqueous humor production and outflow from the anterior chamber predominantly acts to control and maintain IOP in a constant range level. It is now established that most of the aqueous humor outflow occurs through the anterior part of the eye by two different pathways. First, by the trabecular meshwork and the Schlemm’s canal located at the iridocorneal angle which constitute the main route for aqueous humor exit [11]; and second, by the uveoscleral route through the supraciliary body and subciliary muscle spaces [15]. Defects in aqueous humor drainage following either increased flow resistance by the trabeculum or dysfunction of the uveoscleral pathway induce eye hypertonia, which may then favor glaucoma. It has been thought for a long time that there was no flow through the vitreous due to its viscous nature and that there was only passive diffusion exchanges in the posterior eye. However, the reexamination of the existing published data in the literature on this topic in a recent review postulates that this dogma needs probably to be revisited, and that a significant physiological aqueous flow may probably exist through the vitreous, further exiting across the retinal pigmented epithelium and reaching choroidal vessels [16]. On the other hand, fluid movements from the vitreous towards the aqueous humor could also occur, as evidenced by the diffusion and removal of intravitreously injected drugs used to treat diseases that affect the eye posterior segment, through the anterior segment and aqueous humor [17].

## 3. Anatomy of the Eye Lymphatic Vascular System

The presence of lymphatics in the eye region was first identified more than two centuries ago. For details, the reader could refer to the historical considerations reported in the well-documented review written by Grüntzig and Hollmann [6]. However, tremendous advances in the characterization of the eye lymphatic vasculature have been achieved, during the last two decades, following the improvement of imaging techniques and the identification of antigenic markers that discriminate the lymphatic from the blood endothelial cells, such as lymphatic vessel endothelial hyaluronic acid receptor 1 (LYVE-1), podoplanin, vascular endothelial growth factor receptor 3 (VEGFR3) and prospero-related homeobox protein 1 (PROX-1). The existence in mammals of a surface network of ocular lymphatic vessels is clearly established and documented by immunostaining studies that have confirmed and expanded the initial investigations performed by injection of dyes. Lymphatic vessels are present in the corneolimbus bordering the avascular cornea, and in both bulbar and palpebral conjunctiva. This information has been mainly obtained by immunohistological studies on eye sections or on dissected eyes after standard and/or whole-mount immunostainings with lymphatic markers [18,19,20,21]. Other studies performed by direct imaging of eyes from lymphatic fluorescent reporter transgenic animals after either front view or flat-mounting of the anterior eye have more recently confirmed these statements concerning the ocular surface lymphatic vessels distribution [22].

**Figure 2:**
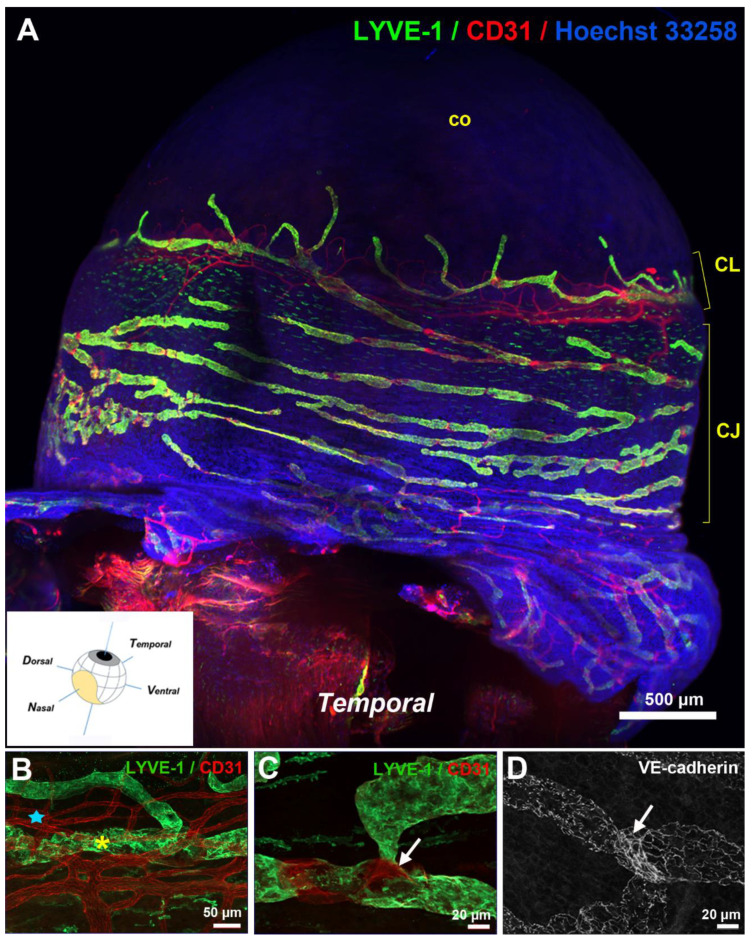
Visualization of the ocular surface lymphatics after whole-mount immunostainings. (**A**) Light sheet fluorescence imaging view of the temporal side of a left mouse eye with LYVE-1 (green) and CD31 (red) antibodies. Nuclei counterstaining was performed with Hoechst 33258 (blue). CJ, conjunctiva; CL, corneolimbus; co, cornea. The inset image, modified from [27], shows a schematic view of the cardinal axes of the left eye and the position of the nictitating membrane (drawn in yellow). (**B**–**D**) Imaging at high magnification of the corneolimbal (**B**) and the conjunctival (**C**,**D**) blood and lymphatic vasculature after whole-mount fluorescence immunostaining and flat mounting of the dissected eye anterior segment. LYVE-1-positive staining marks lymphatic vessels (yellow asterisk) whereas blood vessels (blue star) of the corneolimbus are revealed by the sole CD31-positive staining. The gap in the LYVE-1 staining, as illustrated in (**C**), corresponds to the presence of a valve as revealed by the VE-cadherin staining in another similar field in (**D**). On both images, the valve location is pointed by a white arrow. Note the presence of a more or less punctuated VE-cadherin staining depending on the pre- or the post-valve location, that is typical of lymphatic button- or intermediate button/zipper-type of endothelial cell junctions.

Corneal lymphangiogenesis studies have provided an important source of data on corneolimbal lymphatics [23,24,25]. Corneolimbal lymphatics are LYVE-1-positive and display the conventional oak leaf-like cells with button-like interendothelial junctions. They can develop valves indicating that some of these vessels are likely precollectors [26,27]. Limbal lymphatics have connections with the conjunctival network covering the bulbar conjunctiva. A denser distribution of conjunctival lymphatic vessels at the nasal side of the eyeball was previously reported in a model of induced corneal lymphangiogenesis [28]. The observation of a temporal-nasal polarized distribution has recently been confirmed by innovative techniques, such as LSFM, combined or not with optical clearing of the intact pigmented mouse eyeball, that have allowed the 3D visualization of the lymphatic topography of the whole mouse ocular surface lymphatic network [27,29] (Figure 2).

**Figure 3:**
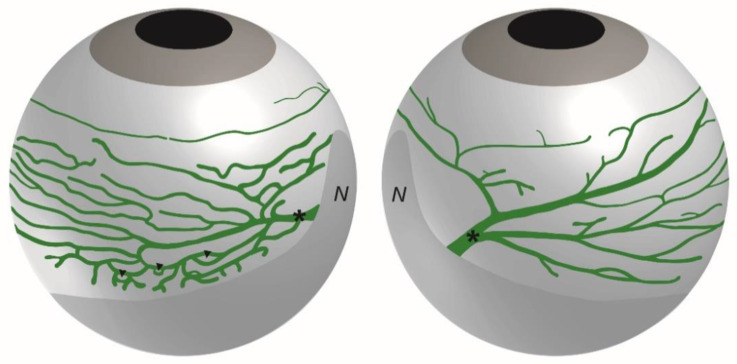
Schematic representation of the lymphatic ocular surface network. The lymphatic network of a right eye on both sides of the nictitating membrane is drawn. The network located at the left side of the nictitating membrane drains the ventral part of the eyeball whereas the network located at the right side of the nictitating membrane drains the dorsal part of the eyeball. The asterisks mark the main draining collecting vessel on each side of the nictitating membrane. Note the typical rich network displaying several loops (arrowheads) at the left side of the nictitating membrane. N, nictitating membrane.

In these studies, the bulbar conjunctiva covering the bulbar sclera, was observed to display a rich network of lymphatic vessels composed of initial lymphatics that drains into LYVE-1-positive precollectors containing valves [27]. As illustrated on the scheme in Figure 3, the ocular surface lymphatics were observed to converge at the nasal canthus by large lymphatic trunks displaying lower LYVE-1 expression and draining either the dorsal or the ventral side of the eyeball [27]. Moreover, these draining lymphatic networks exhibit a different spatial organization on each side of the nictitating membrane depending on the drained ventral or dorsal region of the eyeball, reflecting in addition, a dorsal-ventral polarization (Figure 3). A dense and ramified conjunctival network presenting many circular loops drains the ventral part of the eyeball whereas a less developed vessel network converging in a large individual lymphatic trunk drains the dorsal part of the eyeball [27]. These two different bulbar conjunctival lymphatic networks were symmetrically opposite when comparing the right and the left eyes [27]. The palpebral conjunctival lymphatic vessels also converge at this location in the nasal corner of the eye. The nictitating membrane, also known as the third eyelid, contains cartilage [30,31]. In species where it is fully functional, it can move laterally to cover and protect the eye. The nictitating membrane remains vestigial in rodents and is absent in humans. A representative 3D visualization of the whole surface LYVE-1-positive lymphatic network can be seen on the supplemental Video S1.

The temporal-nasal polarized lymphatic vessel distribution of the conjunctival network was also evidenced in another study examining the organogenesis of these vessels [22]. Eye lymphatic vessel organogenesis was found to occur at early postnatal stages, once the blood vascular plexus has already formed. From these studies, it appears that the ocular surface lymphatic network initially develop from a nascent parent lymphatic vessel present at the base of the nasal canthus by sprouting lymphangiogenesis, further encircling in a concurrent manner both clockwise and counterclockwise the entire eyeball [22]. The question remains of the precise mechanisms involved during the expansion of this network. In addition to sprouting, the involvement of intussusception is well established during angiogenesis [32]. During this process, new blood vessel formation results from transcapillary pillar formation and further parent vessel splitting. Intussusceptive lymphangiogenesis has recently been reported in lymphatic malformations observed in lymphangioma and in the formation of lymph node sinuses during lymph node development [33,34]. We can assumed that a similar mechanism could also contribute to the expansion of the eye lymphatic network. On the other hand, it may also been asked whether part of the network could develop from single lymphatic clusters that may further connect between themselves. It is now established that not all of the lymphatics differentiate from their venous counterparts and that non-venous cellular origins could also exist, depending on the tissue considered [35,36].

With regard to the inner eye, the existence of traditional lymphatics has been highly debated. Some podoplanin-positive and/or LYVE-1-positive channels were identified in the ciliary bodies in human and sheep [37]. However, these data remain controversial since they were not confirmed by other groups [38,39]. In both human and mouse, these ciliary body LYVE-1-positive cells were reported to belong to the macrophage lineage [27,39,40]. Similar conclusions can be drawn for the iris [38,39] and the choroid [41,42]. Despite the presence of cells expressing some lymphatic markers, immunohistological studies have revealed that these markers were not coexpressed by the same cell types. In addition to lymphatic endothelial cells, LYVE-1 immunostaining is largely distributed in most of the eye internal and external tissues including the optic nerve. The observed LYVE-1-positive cells remained either dispersed or close to blood capillaries exhibiting the characteristic features of paravascular macrophages [27,40,43,44]. All these observations have supported the conclusion that classical lymphatic vessels are absent in internal uveal tissues and in posterior eye in physiological conditions. However, the question of the existence of an uncommon LYVE-1-negative organ-specific lymphatic vessel subtype with a specific gene expression pattern, and/or the existence of interspecies differences in the eye lymphatic vasculature, cannot be fully excluded [27]. Most of the studies concluding to the lack of uveal lymphatics were performed in rodents whereas studies postulating their presence were performed in sheep and humans. In our opinion, the possible existence of such an organ- and/or species-specific lymphatics in internal eye structure should be further evaluated.

## 4. Molecular Mechanisms Regulating Eye Lymphatic Vessel Development

It could be expected that the development of the eye lymphatic vasculature mobilizes some common signaling pathways described to regulate lymphatic development in other organs. In mammals, lymphatic vascular development initiates at mid-gestational embryonic developmental stages (from approximately E9.5 in the mouse) from the wall of the cardinal vein [45]. The differentiation of the lymphatic endothelial lineage from a subpopulation of venous endothelial cells is controlled by the coordinated expression of several transcription factors including SOX18 (sex determining region Y-box 18), COUP-TFII (chicken ovalbumin upstream promoter transcription factor 2), HHEX (hematopoietically expressed homeobox) and GATA-2 (GATA binding factor 2). These transcription factors cooperate to activate PROX-1, the key transcription factor constituting the master gene for lymphatic endothelial specification and lymphatic identity maintenance. The budding of the specified lymphatic endothelial cells leading to the formation of lymph sacs and the further expansion of a functional lymphatic vasculature are under the control of the VEGFC/VEGFR3 signaling pathway, the major lymphangiogenesis regulator in vertebrates [45]. Another important molecular signaling pathway which participates in the remodeling and the maturation of the lymphatic vascular system is constituted by the angiopoietins/TIE receptors system [46]. Angiopoietin-1 and angiopoietin-2 binding to the receptor TIE-2 regulate both blood and lymphatic vascular development [46,47]. In some organs, such as the heart, lymphatic vessels have been shown to have a dual venous and non-venous origin and to form later during postnatal steps, highlighting the existence of some organ-specific differences in the cellular origins and in the regulatory mechanisms during lymphatic vascular system development [35]. Interestingly, the Schlemm’s canal, a highly specialized vessel which constitutes the main pathway for aqueous humor outflow, present a blood venous and lymphatic hybrid identity. It displays expression of some lymphatic molecular markers such as PROX-1 and VEGFR3 and shares regulatory mechanisms with those involved in lymphatic vessel development [48]. Indeed, the two major VEGFC/VEGFR3 and angiopoietin/TIE signaling pathways were found essential for Schlemm’s canal formation and/or maintenance.

In this review, we will focus our attention on classical ocular lymphatic vessel formation. As expected, the main lymphangiogenesis regulatory signaling pathway comprising VEGFC and its receptor VEGFR3 is critical. VEGFC was found essential for limbal lymphatic vessel formation during development. Indeed, limbal lymphatics were nearly absent in heterozygous VEGFC-deleted mutant mice at postnatal stage P7 [49,50], and their formation was greatly inhibited in wild type mice that were administered with soluble VEGFR3 (sVEGFR3) between P1 and P6 [50]. A second important pathway for ocular lymphatic development is constituted by the angiopoietins/TIE-2 signaling pathway. A major work from Susan Quaggin’s group have identified this signaling pathway critical for ocular lymphatic vessel formation in addition to Schlemm’s canal development. In transgenic mice, conditional deletion at late embryonic stages (after E16.5) of both angiopoietin-1 and angiopoietin-2, resulted in the failure to develop eye lymphatics [51]. A compensatory effect between both ligands has been evidenced by the lack of lymphatic defects if only single conditional deletion of either angiopoietin-1 or angiopoietin-2 alone was performed [51]. In the future, it may be interesting to check if angiopoietin-4, another identified ligand for endothelial receptor tyrosine kinase TIE-2, which appear involved in venous development [52], may participate in the process.

In addition to their developmental effects, it appears important to mention that these pathways also participate in the regulation of the ocular lymphatic vasculature once it has formed and/or in pathological situations. Indeed, VEGFC is also a major regulator for corneal lymphangiogenesis [53], and its blockade was reported in several studies to inhibit the inflammation-induced cornea invasion by lymphatic vessels [54]. VEGFC may originate from macrophages present in the limbus, which have been shown to be important contributors for inflammation-induced corneal lymphangiogenesis [55,56]. Consistent with an essential role for VEGFC, sVEGFR3 secreted by the corneal epithelium was shown to participate to the maintenance of the cornea transparency by inhibiting the lymphangiogenesis response [57]. On the other hand, angiopoietin-2 can also induce corneal lymphangiogenesis from the established corneolimbal lymphatic vasculature and angiopoietin-2 blockade was reported to favor corneal graft survival [58].

Bone morphogenetic protein 9 (BMP9)-activated signaling pathway has recently been evidenced to constitute a third regulatory pathway involved in eye lymphatic maturation [27]. BMP9 is a blood circulating factor secreted by the liver, member of the transforming growth factor β (TGFβ) superfamily, which constitutes a high affinity ligand for the ALK1 receptor, mainly expressed on endothelial cells [59]. It was characterized in vivo to act as a blood vascular quiescence factor, and to control with BMP10, the blood retinal vascularization at postnatal developmental stages. *Bmp9* gene deficiency was also previously reported to selectively affect the lymphatic vasculature maturation process and valve formation associated with reduction in lymphatic drainage efficiency, but without inducing any defects in blood vessels [59]. As previously observed in other tissues, a significant reduction in valve numbers was measured in eye lymphatic vessels of the conjunctiva [27]. In addition, enlargement of the main lymphatic vessels draining the dorsal part of the eyeball was observed, thus allowing to expect some differences in drainage efficiency. However, in contrast to the fore-mentioned VEGFC and angiopoietins signaling pathways, the BMP9-activated pathway only affects lymphatic vessels without any significant repercussions on the Schlemm’s canal morphogenesis [27]. This may result from the Schlemm’s canal vessel dual phenotype since it presents a hybrid blood and lymphatic vessel identity. These data support a selective role for the BMP9-activated signaling pathway on lymphatic vessels that may constitute an interesting way for the specific targeting of ocular lymphatics without interacting with the Schlemm’s canal. With regard to the specific process of valve formation and maintenance, Ephrin B2/EPHB4 signaling was also observed to be involved in eye lymphatics, as previously characterized in the lymphatics of the mesentery [60].

## 5. Functional Role of Eye Lymphatics in Aqueous Humor Drainage and Intraocular Pressure

Ocular surface lymphatics could exert several critical roles. Lymphatic vessels constitute the route for antigen-presenting cell trafficking to draining lymph nodes, and are responsible for the induction of adaptive immunity [1,2]. Reciprocal interactions between lymphatic endothelial cells and immune cells have been described [61]. Corneal lymphangiogenesis can develop from the limbal lymphatic network and can invade the cornea, after injury or in ocular inflammatory diseases [62,63]. The lymphangiogenic response allows the control of inflammation and contributes to favor its resolution, by capturing extravasated fluids from leaky blood vessels and by eliminating cell debris and pathogens [64]. In this context, ocular lymphatics have been described to regulate corneal edema following eye injury [65]. In contrast, the immune response may be inappropriate in some chronic inflammatory pathological situations such as dry eye disease, where antilymphangiogenic therapeutic strategies have been postulated beneficial [63]. Lymphangiogenesis inhibition also constitutes a prerequisite to favor corneal graft survival and to prevent rejection during cornea transplantation [66]. Several well-documented reviews, which the reader could refer to, have been published on the topic of lymphatic vessel involvement in ocular inflammatory diseases [5,25,67]. Moreover, considering the lymphatic vessel essential role in tissue fluid homeostasis, ocular lymphatics may also overall contribute to the regulation of ocular fluids. In this review, we will focus our attention on their potential role in aqueous humor drainage, a major process whose dysfunction may lead to intraocular pressure increase, a major risk factor for glaucoma [14].

**Figure 4:**
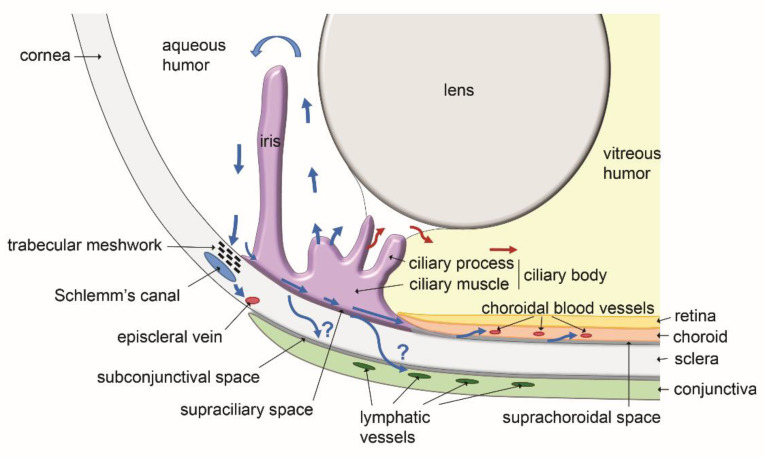
Schematic representation of the routes of aqueous humor transport in the ocular anterior region (blue arrows: outflow; red arrows: inflow). Aqueous humor is produced by the ciliary processes. Although some inflow could occur towards the posterior region of the eye, most of the aqueous humor flows in the anterior chamber and exits through the iridocorneal angle. Two major drainage pathways are well characterized (blue arrows): the conventional trabecular meshwork and Schlemm’s canal pathway, and the uveoscleral non-conventional route through the supraciliary and the suprachoroidal spaces. By both of these pathways, aqueous humor reaches the blood venous circulation in either episcleral veins or choroidal vessels, respectively. A third route using the subconjunctival space and/or the lymphatic vessels of the conjunctiva also appears to exist, draining aqueous humor to the neck lymph nodes. Scheme inspired from [14].

Aqueous humor is known to leave the eye through two major pathways. The direct conventional route, constituted by the trabeculum and the Schlemm’s canal, and the indirect unconventional route involving the ciliary body and the interstitial spaces of the ciliary muscle and the suprachoroidal space [15]. These pathways, with in addition the putative lymphatic routes discussed below, are schematically illustrated on Figure 4. The involvement of ocular lymphatics as a part of the unconventional aqueous humor outflow has been proposed following observations reporting the diffusion of intracamerally injected tracers to lymph nodes of the neck in several experiments performed with animals. Radioactive tracers injected in the rabbit eye anterior chamber were reported to accumulate into the superficial cervical lymph nodes [6]. A drainage connection between the rabbit eye anterior chamber and the lymphatic system was also supported by the observation that cervical lymph flow blockade by surgery or ligature significantly reduced the tracer diffusion [6].

In other studies, intracameral injection of fluorescent-labelled antigens in rat eyes was also found to result in their accumulation in lymph nodes of the head and the neck, at 24 h post-injection [68]. Although a large proportion of the injected tracer entered the venous circulation through the conventional route, the authors hypothesized the existence of a conjunctival lymphatic route. Several recent studies of intracameral tracer injection performed in mice further support the involvement of lymphatics for the injected tracer to reach the cervical lymph nodes. This information has been obtained by different non-invasive imaging techniques for the detection of either radioactive tracers or fluorescent dyes, or by photoacoustic tomography [69,70,71,72]. The existence of a conjunctival lymphatic drainage route was also obtained in rats when tracer injection was performed in the subconjunctival space. This ocular surface lymphatic drainage route observed in rats [68,73], was recently confirmed in mouse when using photoacoustic tomography imaging to follow the diffusion of subconjunctivally injected tracer [74]. Such a route was also evidenced in humans after glaucoma filtration surgery [75].

The pathway for aqueous humor drainage to lymph nodes has also been postulated to initiate through ciliary body lymphatics. This hypothesis originates from a study performed in the sheep which showed that intracamerally injected fluorescent nanospheres can be visualized a few hours later, in the lumen of a LYVE-1-positive ciliary body lymphatic vessel, on eye cryosections [37]. However, since the existence of conventional lymphatic vessels in the internal eye structures has not been confirmed by different groups and remains highly controversial, the ciliary body lymphatic route assumption should be taken with caution. On the other hand, a direct absorption of aqueous humor by corneolimbal and/or conjunctival lymphatics has not been clearly demonstrated. With regard to a possible aqueous humor outflow through conjunctival lymphatics, anatomical studies have not described until yet, the presence of trans-scleral lymphatics [43]. Nevertheless, a passive trans-scleral diffusion of aqueous humor may occur [76].

Then, the question of the importance of the lymphatic route in aqueous humor drainage still remains. In accordance with a minor contribution of eye lymphatics in aqueous humor drainage, the eye conjunctival lymphatic defects induced by *Bmp9* gene deficiency in mice were without repercussions on IOP. This information was obtained in both control normal conditions or when the mice were challenged in a reversible hypertonia-induced model, thus questioning for a potential conjunctival lymphatic vessel role in aqueous humor drainage and IOP regulation, unless in the case of very severe lymphatic defects [27]. Moreover, the aqueous humor drainage by lymphatics is not consistent with the recently described data reporting inhibition of newly formed lymphatics and/or lymphatic vessel regression of pre-established lymphatic vessels by aqueous humor components [77,78]. If antilymphangiogenic factors are present in the aqueous humor, and if aqueous humor drains through lymphatic vessels, these vessels would probably not be able to support efficiently drainage, unless there may exist different lymphatic vessel subtypes displaying different aqueous humor-contained antilymphangiogenic factors sensitivities, avoiding their regression. In any case, the contribution of lymphatics to aqueous humor drainage may not be very substantial. After examination of the quantitative studies performed after intracamerally injected radioactive albumin tracer, it appeared to represent only a minor part of the total aqueous humor outflow [79]. Consistent with these data, the observed reduction in the aqueous humor lymphatic drainage when comparing young and aged mice was not associated with significant variations in IOP values, which remained unchanged [80].

However, in support of a lymphatic role in aqueous humor drainage, the effect of Latanoprost in the reduction of IOP was no longer observed after cervical lymph node dissection [81]. This showed that the disruption of the lymphatic vessel lymph node endpoint could interfere with aqueous humor outflow and IOP. The splitting of the aqueous humor outflow through different routes reaching lymph nodes have been evidenced in rat eyes [82]. Several lymphatic drainage pathways have also been described in the mouse head [83]. This may generate differences in the observations made, in particular concerning the timing and the final lymph node location of the tracer diffusion. At the difference of intracameral tracer injection which mostly resulted at final time endpoints in accumulation in submandibular and deep cervical lymph nodes, subconjunctival injection lead in addition to tracer accumulation in superficial parotid lymph nodes [72]. These different routes could also be differently followed in a time-dependent manner. Since the importance of the unconventional uveoscleral aqueous humor drainage pathway, within it lymphatics are postulated to constitute a part, was reported to vary among species [76], aqueous humor lymphatic drainage may thus be also differently involved according to the species considered.

## 6. Interaction of an Ocular Glymphatic System with IOP and Retinal Fluid Clearance

Despite the lack of evidence for a significant impact on IOP, lymphatics may participate in connection with an ocular glymphatic (glial lymphatic) system in the clearance of metabolites from the eye. This may be of particular importance since the high metabolic activity of the retina generates several waste products comprising neurotoxic proteins that need to be properly cleared. Indeed, dysfunction in the clearance of waste metabolic products from the posterior eye may cause damage to the retina and may be involved in retinal eye diseases associated with neurodegeneration [84,85]. The retina lacks a conventional lymphatic drainage system [86] but an eye glymphatic system may participate in this cleaning process. Indeed, during the previous years, the contribution of an ocular glymphatic system similar to the one present in the brain, has been proposed in the regulation of the posterior eye fluid homeostasis [87,88]. The retina covering the posterior segment of the eye is considered as a projection of the brain. Retinal ganglion cell axons extend outside the retina and converge in the optic nerve fibers to connect with neuronal cells in the brain [89,90]. In the central nervous system, a meningeal lymphatic vascular system [91], appears to be interconnected with a brain parenchymal glymphatic fluid transport system, which corresponds to the network of paravascular spaces that are formed by astrocytes endfeet surrounding the blood vessels constituting the blood brain barrier [92]. Aquaporin water channels, and in particular aquaporin-4, have been postulated as elements of the astrocyte endfeet allowing cerebrospinal fluid (CSF) and brain interstitial fluid (ISF) exchanges by convective flow from para-arterial to paravenous spaces, favored by a pressure gradient caused by the arterial pulsations [93,94,95]. Despite controversies concerning the real contribution of aquaporin-4 and the exact mechanisms and routes involved [96,97,98], this fluid transport appears important for the clearance of solutes and waste metabolic products from the CSF and ISF, allowing the brain homeostasis. Moreover, drainage of CSF to deep cervical lymph nodes has been reported in both rodents and humans, thus contributing to the clearance of waste products from the brain [99,100,101].

**Figure 5:**
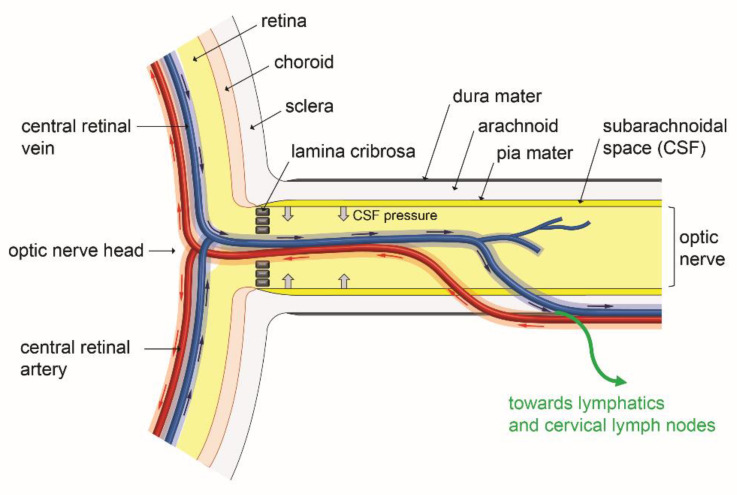
Overview of glymphatic system involvement in fluid transport in the posterior eye. Para-arterial influx (red arrows) and paravenous efflux (blue arrows) occurring in the paravascular spaces of the main retinal artery and vein may respectively bring solutes and eliminate waste products in the eye retinal layers. After further transport in the paravascular spaces of the optic nerve vein, waste metabolic products may enter true lymphatics and move towards cervical lymph nodes. These fluid movements may be affected by the hydrostatic pressure of CSF (cerebrospinal fluid) which is present in the subarachnoidal space and which could exert mechanical forces (large gray arrows) interfering with the paravascular fluid circulation in the glymphatic system. Scheme inspired from [88,104,105].

In addition to several hypothesis made during the previous years, more evidence for the existence of a paravascular glymphatic system in the eye, and in the optic nerve in particular, has been obtained in a recent investigation [102]. Based on the imaging of the diffusion of intravitreously administered fluorescent amyloid-β as a tracer, this work provided arguments suggesting the existence of a polarized clearance system in the posterior segment of the eye and along the optic nerve. The tracer was found to be rapidly transported along the paravascular spaces of the optic nerve veins. Moreover, it can also reached cervical lymph nodes suggesting the possible involvement of lymphatics [102]. Figure 5 schematically illustrates the involvement of the paravascular glymphatic system in eye fluid transport in the eye posterior segment. If confirmed by additional experiments, the existence of such a system may provide to the retina a clearance pathway for the neurotoxic amyloid-β protein, which can accumulate in lipid- and protein-rich deposits constituting the drusen, responsible for the development of age-related macular degeneration (AMD) [89]. Significant deposition of the amyloid-β neurotoxic protein has also been postulated to cause retinal ganglion cell death in glaucoma [88]. In addition, several works mentioned extracellular accumulation of glutamate in retinal ganglion cell synapses that may also exert toxic effects on these cells [103].

The newly described glymphatic system could probably be involved in fluid drainage and neurotoxic product clearance from the internal posterior part of the eye. In this context and as observed in cerebral small vessel disease, enlarged paravascular spaces could reflect stasis and a defect in the clearance of waste products [106,107,108]. Molecular regulation of aquaporin-4 water channel expression may then be interesting to consider to modulate fluid movement in this paravascular compartment, and could be at the basis of new therapeutic strategies for neuroprotection in eye diseases. Such a proposal was already done in brain neurodegenerative diseases [95]. Interestingly, the intravitreally injected amyloid-β clearance appeared reduced in aquaporin-4 deficient mice [102]. It can then be expected that increased aquaporin-4 expression may favor water exchange and clearance of waste products by glymphatic drainage along the optic nerve. Targeting aquaporin-1 and/or aquaporin-4, which are expressed in ocular tissues, have also been proposed for improvement of ocular fluid dynamics in glaucoma [109]. However, the examination of the real potential benefit effects of aquaporin modulators of expression in this disease should be carefully examined since aquaporins are also involved in the aqueous humor secretion process [110], and in front of the remaining discrepancies about their role in the glymphatic system [96,97,98].

It is also clear that the hypertonia is only one component for the progression of glaucoma that also relies on retinal ganglion cell degeneration which can result from the drainage defect of neurotoxic waste products [88]. In this later clearance process, the eye glymphatic system is expected to further connect with lymphatic vessels, and both systems may then be essential for eye neuroprotection. Consistent with this statement, glymphatic activity is described to decrease with aging [111,112], which coincides with the appearance of eye neurodegenerative diseases in the elderly persons. However, the real importance of the glymphatic outflow in amyloid-β transport in glaucoma should be further explored since intravitreously injected amyloid-β tracer movements along the optic nerve were found increased in two mouse glaucoma models [102]. On the other hand, the development of glaucoma may be influenced by the CSF pressure since the paravascular spaces of the posterior eye may be affected by the pressure of the CSF fluid surrounding the optic nerve [113]. Such an interaction could occur in the anatomic region of the lamina cribrosa, located at the basis of the optic nerve head, which forms the interface between the intraocular and the retrobulbar compartments [113,114]. Recent investigations support the interaction between the cerebral pressure and the para-arterial inflow from the CSF to the optic nerve [115,116,117]. The lamina cribrosa region appears to constitute a critical site subjected to the pressure gradient resulting from both CSF pressure and aqueous humor-induced IOP. As illustrated on Figure 5, the opposite forces resulting from the CSF surrounding the optic nerve may interfere with the glymphatic fluid flow exiting the eye and with waste drainage, that could ultimately result in the damaging of retinal ganglion cells and optic nerve fibers, contributing to glaucoma. However, the different routes involved in amyloid-β clearance should be clarified since an amyloid-β intra-axonal transport, awaiting to be clearly demonstrated, has also been postulated during its exit through the lamina cribrosa [102].

## 7. Concluding Remarks

Important progress has been made in the last decade concerning the knowledge of lymphatic and glymphatic systems involvement in ocular physiopathology. However, there are several remaining questions that should be answered for a better insight in their functions and in the interest for their therapeutic targeting.

Improvements in methods for the exploration of the glymphatic function as well as defining strategies to stimulate the fluid movement in this paravascular compartment should bring novel significant advances. Particular attention should also be given in future studies to the analysis of the lymphatic vasculature in human eyes to validate the results obtained in rodent models. The drainage of aqueous humor by lymphatics remains not unambiguously demonstrated. It would be important to solve the gap in the knowledge of how aqueous humor can reach the lymphatics in physiological conditions and to clearly identify the different routes by which aqueous humor could enter into the lymphatic vasculature and reach lymph nodes. Moreover, it also appears essential to establish whether targeting the ocular lymphatics may have a significant functional consequence in aqueous humor drainage and further IOP regulation. On the other hand, one has to keep in mind that targeting lymphatics may also affect Schlemm’s canal, depending of which signaling pathway is considered. This may constitute an adverse effect, especially when lymphangiogenesis inhibition is needed in the context of eye inflammatory diseases or corneal transplantation. Further analysis of the involvement of the glymphatic and lymphatic systems in the clearance process of waste products from the eye should also be performed, since these systems may constitute new targets in therapeutic strategies designed to fight against eye neurodegenerative diseases such as AMD and glaucoma.

## Data Availability

Not applicable.

## Supplementary Materials

The following are available online at https://www.mdpi.com/article/10.3390/biology10070582/s1. Video S1: 3D visualization of the eye surface lymphatic network.
