# Supplementary material for: Lymphatics in Eye Fluid Homeostasis: Minor Contributors or Significant Actors?
Source: Biology (Basel). 2021 Jun 25;10(7):582. doi: 10.3390/biology10070582 (PMC8301034; doi:10.3390/biology10070582)
Supplement: Supplementary file 1 [file biology-10-00582-s001.zip › 210518_Supplemental Information_Subileau&Vittet.pdf]

Lymphatics in eye fluid homeostasis: minor contributors or significant actors?

Mariela Subileau[1], Daniel Vittet[1*]

[1]University of Grenoble Alpes, Inserm, CEA, IRIG-DS-BIOSANTE, 38000 Grenoble, France

*Contact: daniel.vittet@cea.fr

Supplemental Information:

Video S1: 3D visualization of the eye surface lymphatic network.

Representative movie allowing the 3D visualization of the whole surface LYVE-1-positive lymphatic network of a right mouse eye. This video depicts a clockwise view of the surface lymphatic vasculature, starting left to the nictitating membrane. Note that part of the ventral palpebral conjunctival lymphatic network can be seen at the basis of the eye.
